# Blueberry Consumption Challenges Hepatic Mitochondrial Bioenergetics and Elicits Transcriptomics Reprogramming in Healthy Wistar Rats

**DOI:** 10.3390/pharmaceutics12111094

**Published:** 2020-11-14

**Authors:** Sara Nunes, Sofia D. Viana, Inês Preguiça, André Alves, Rosa Fernandes, João S. Teodoro, Artur Figueirinha, Lígia Salgueiro, Sara Silva, Ivana Jarak, Rui A. Carvalho, Cláudia Cavadas, Anabela P. Rolo, Carlos M. Palmeira, Maria M. Pintado, Flávio Reis

**Affiliations:** 1 Institute of Pharmacology & Experimental Therapeutics & Coimbra Institute for Clinical and Biomedical Research (iCBR), Faculty of Medicine, University of Coimbra, 3000-548 Coimbra, Portugal; sara_nunes20@hotmail.com (S.N.); sofia_viana@estescoimbra.pt (S.D.V.); i.preguica@campus.fct.unl.pt (I.P.); alves.andrefb@gmail.com (A.A.); rcfernandes@fmed.uc.pt (R.F.); 2 Center for Innovative Biomedicine and Biotechnology (CIBB), University of Coimbra, 3004-504 Coimbra, Portugal; ccavadas@uc.pt; 3 Clinical Academic Center of Coimbra (CACC), 3004-504 Coimbra, Portugal; 4 Polytechnic Institute of Coimbra, ESTESC-Coimbra Health School, Pharmacy/Biomedical Laboratory Sciences, 3046-854 Coimbra, Portugal; 5 Department of Life Sciences, Faculty of Science and Technology (FCTUC), University of Coimbra, 3000-456 Coimbra, Portugal; jteodoro@ci.uc.pt (J.S.T.); rac@uc.pt (R.A.C.); anpiro@ci.uc.pt (A.P.R.); palmeira@uc.pt (C.M.P.); 6 Center for Neurosciences and Cell Biology of Coimbra (CNC), University of Coimbra, 3004-504 Coimbra, Portugal; 7 Faculty of Pharmacy, University of Coimbra, 3000-548 Coimbra, Portugal; amfigueirinha@ff.uc.pt (A.F.); ligia@ff.uc.pt (L.S.); 8 LAQV, REQUIMTE, Faculty of Pharmacy, University of Coimbra, 3000-456 Coimbra, Portugal; 9 CIEPQPF, Chemical Process Engineering and Forest Products Research Centre, University of Coimbra, 3000-456 Coimbra, Portugal; 10 CBQF—Centro de Biotecnologia e Química Fina—Laboratório Associado, Universidade Católica Portuguesa, Escola Superior de Biotecnologia, Rua Diogo Botelho 1327, 4169-005 Porto, Portugal; snsilva@porto.ucp.pt (S.S.); mpintado@porto.ucp.pt (M.M.P.); 11 Department of Microscopy, Laboratory of Cell Biology and Unit for Multidisciplinary Research in Biomedicine (UMIB), Institute of Biomedical Sciences Abel Salazar (ICBAS), University of Porto, 4050-313 Porto, Portugal; jarak.ivana@gmail.com; 12 Associated Laboratory for Green Chemistry-Clean Technologies and Processes, REQUIMTE, Faculty of Sciences and Technology, University of Porto, 4050-313 Porto, Portugal

**Keywords:** blueberries, long-term consumption, bioenergetics remodeling, transcriptomics reprogramming, anti-inflammatory pre-conditioning

## Abstract

An emergent trend of blueberries’ (BB) “prophylactic” consumption, due to their phytochemicals’ richness and well-known health-promoting claims, is widely scaled-up. However, the benefits arising from BB indiscriminate intake remains puzzling based on incongruent preclinical and human data. To provide a more in-depth elucidation and support towards a healthier and safer consumption, we conducted a translation-minded experimental study in healthy Wistar rats that consumed BB in a juice form (25 g/kg body weight (BW)/day; 14 weeks’ protocol). Particular attention was paid to the physiological adaptations succeeding in the gut and liver tissues regarding the acknowledged BB-induced metabolic benefits. Systemically, BB boosted serum antioxidant activity and repressed the circulating levels of 3-hydroxybutyrate (3-HB) ketone bodies and 3-HB/acetoacetate ratio. Moreover, BB elicited increased fecal succinic acid levels without major changes on gut microbiota (GM) composition and gut ultra-structural organization. Remarkably, an accentuated hepatic mitochondrial bioenergetic challenge, ensuing metabolic transcriptomic reprogramming along with a concerted anti-inflammatory pre-conditioning, was clearly detected upon long-term consumption of BB phytochemicals. Altogether, the results disclosed herein portray a quiescent mitochondrial-related metabolomics and hint for a unified adaptive response to this nutritional challenge. The beneficial or noxious consequences arising from this dietary trend should be carefully interpreted and necessarily claims future research.

## 1. Introduction

The continuous rise in life expectancy observed in the last decades encloses an ascending trajectory of non-communicable diseases (NCDs), which are often linked with unhealthy dietary patterns [1,2]. Society is progressively becoming more aware of healthy eating to prevent diet-related chronic diseases. New trends in food consumption have fostered agro-food and pharmaceutical companies to develop new products, both wellness-focused diets, functional foods, and oral nutraceutical supplements, to meet consumer demands [3]. A good example of this reality is blueberry (BB) market globalization, whose per capita consumption nearly tripled in USA since 2002 and is fast-expanding worldwide [4,5]. 

The low caloric content of BB (0.046 kcal/g fresh fruit) pair with their enriched nutritional and phytochemical composition [6]. BB is a privileged source of micronutrients (e.g., selenium, zinc, iron), dietary prebiotic fibers (3–3.5% of their fruit weight), and antioxidant polyphenols encompassing anthocyanins, flavonols, phenolic acids, procyanidins, and/or stilbenes derivatives, with an overall content reaching up to 0.3% of fresh fruit weight [6,7]. These bioactive compounds are extensively metabolized by the colonic microbiota; in addition, regardless of their poor oral bioavailability [8,9,10], BB-derived phytochemicals can positively modulate chief endogenous functions in distinct organs and tissues that extend beyond their well-documented antioxidant properties [11,12,13,14]. In fact, experimental data arising from cell-free systems, standard cell-cultures, and/or isolated organelles highlight BB phytochemicals’ ability to modulate non-redox mechanisms through their interactions with functionally diverse cellular targets, such as intercalation with DNA, transcription of several genes associated with key cellular functions, mitochondria dynamics, and even gut microbiota (GM) homeostasis [4,15,16,17,18]. Convergent results are also reported in preclinical animal studies that emphasize BB consumption benefits in a panoply of chronic disorders paralleling obesity-related metabolic diseases such as cardiovascular disease (CVD), metabolic syndrome (MS) or type 2 diabetes mellitus (T2DM) [13,19,20,21,22,23,24,25]. Interestingly, the association between human BB consumption and biomarker-based evidence of reduced risk of diseases has been also emphasized [13,26,27,28,29,30]. In fact, several clinical trials have progressively emphasized on potential health benefits (e.g., endothelial, gastrointestinal, cardio-metabolic outcomes) arising from long-term BB-enriched human dietary patterns and commercially available BB supplements with the ultimate goal to empower overall health status [31,32,33,34,35]. Regardless of this wealth of evidence, incongruent preclinical and human data still remain an open debate and the translation into the clinical practice remains puzzling, mainly due to critical flaws surrounding studies’ reproducibility [29,30,34,36,37]. For instance, in vitro assays often employ high-concentrations of BB-derived phytochemicals that are unlikely to reach systemic circulation once orally ingested in living organisms [38]. Besides dose, the compounds’ bioactivity is often dependent on (i) how an organism is exposed to, (ii) for how long, and (iii) interindividual variability. The heterogeneity surrounding BB-derived phytochemicals’ intake regarding the presentation forms (e.g., fresh/frozen/freeze-dried fruit, distinct cultivars, the range of commercially available supplementation forms) and regimen durations (e.g., short- versus long-term) actually convolutes the interpretation of contemporary preclinical and human data. Moreover, the high interindividual human variability is also well-recognized in terms of BB phytochemicals’ bioavailability and bioactivity, which is largely dependent of the individual gut microbiome [3,26,29,34,39]. Thus, it is currently challenging to judge the benefits and/or hazardous consequences underlying this nutraceutical contemporary trend, which necessarily calls for further research.

To this end, we conducted a translation-minded experimental design in young adult healthy Wistar rats who were daily supplemented with a dose of 25 g/kg of whole fresh BB, mirroring recent clinical trials [34,39,40,41] in a long-term regimen (14 weeks) [42]. Particular attention was paid to the physiological adaptations ensuing in the gut and liver tissues regarding their well-known metabolic chief functions.

## 2. Materials and Methods 

### 2.1. Preparation of Blueberry Juice 

Blueberries (*Vaccinium corymbosum* L., cultivar “Liberty”) were provided from the same variety and in the same maturation stage by COAPE (Farming Cooperative of Mangualde, Mangualde, Portugal) and stored at −80 °C until processing. To ensure that whole parts of BB fruits (peel, pulp, and seeds) were consumed, the BB were weighed, blended with drinking water, and transformed into BB juice (BJ). The amount of drinking water added was adjusted to ensure that 25 g of BB (per kg of rat’s BW) were daily consumed. This BB dose was based on previous preclinical and human studies [34,40,43], and is equivalent to a daily consumption of 240 g of fresh whole BB (approximately 3/2 daily cups of BB), established taking into account the surface area of a person weighing ≈60 kg [40,44]. Since whole BB juice loses about 83% of its anthocyanins content and about 40% of its antioxidant activity during storage at 4–8 °C for 10 days, BJ was freshly prepared on a daily basis [45].

### 2.2. Phytochemical Analyses of Phenolic Compounds in Blueberry Juice

A sample of fresh BJ was concentrated under reduced pressure and freeze-dried for phytochemical analysis. The lyophilized juice was dissolved in water (7 mg/mL) and injected (100 µL) in a High performance liquid chromatography (HPLC) (Gilson, Middleton, WI, USA) hyphenated with a photodiode array detector PDA (model 170) and a control and processing software (Unipoint^®^ 2.10). The chromatograph was equipped with an auto sampler (234 autoinjector), two pumps (models 305 and 306), a manometric module (model 805), a mixer (Model 811 B), and an C18 analytical column (Spherisorb Waters^®^ S5 ODS2; 250 × 4.6 mm, 5 μm particle size), maintained at 35 °C, preceded by a guard column KS 30/4 Nucleosil 120–5 C-18, Macherey-Nagel (Duren, Germany). A mixture of 5% aqueous formic acid solution (A) and methanol (B) was used as mobile phase, with gradient elution of 0–75 min (0–100% B) at a flow rate of 1 mL/min. The UV-vis spectra were obtained between 200 and 600 nm.

### 2.3. Animals and Experimental Design

Male Wistar rats (16-weeks-old) were purchased from Charles River Laboratories (Barcelona, Spain) and housed two per cage in ventilated cages, with controlled environmental conditions (22 ± 1 °C, relative humidity of 50–60% and a 12 h light-dark cycle) and ad libitum access to standard rodent chow and tap water. After one week of acclimatization period, rats were randomly assigned into two groups (*n* = 8 per group): (i) Control group (CTRL), maintained with standard rat chow containing 8.6% kcal from fat (4RF21, Mucedola^®^, Milan, Italy) and tap water and (ii) Blueberry juice group (BJ), fed the same standard chow and supplemented daily, during the experimental period of 14 weeks, with 25 g/kg body weight (BW)/day of BJ. Following the daily dose of BJ intake, drinking water was provided ad libitum. 

The experiment was carried out in strict compliance with the National and European Communities Council Directives of Animal Care and with the ARRIVE guidelines for reporting animal research [46]. The animal’s protocol was approved by the local (iCBR) Animal Welfare Body (ORBEA, #9/2018, 30 October 2018).

Feed and beverage were provided ad libitum, with exception of the fasting periods. Beverage volume were BW was monitored weekly; food and beverage consumption were daily recorded per cage throughout the experimental protocol. Energy intake per week was calculated for each animal by using the former measurements.

### 2.4. Glucose Tolerance Test (GTT) and Insulin Tolerance Test (ITT)

During the first days of weeks 13 and 14, GTT and ITT were performed to assess the rats’ ability to tolerate a glucose load and to evaluate peripheral insulin sensitivity, respectively, as previously described [47]. For the GTT, after 6 h of fasting period (between 8:00 a.m. and 2:00 p.m.), conscious rats were intraperitoneally (i.p.) injected with a glucose solution of 2 g/kg BW and blood glucose (BG) levels were measured from the tail blood, recorded immediately before (0 min) and 30, 60, and 120 min after injection, using a portable glucometer (ACCU-CHEK^®^ Aviva, Roche Diagnostics, Mannheim, Germany). Additionally, blood samples (≈30 μL) were collected before glucose challenge to determine the fasting insulin concentration.

For the ITT, animals were fasted during 6 h and injected i.p. with insulin solution (0.75 units/kg BW; Actrapid Novo Nordisk, Bagsvaerd, Denmark). Blood glucose levels were measured in the tail vein blood collected immediately before (0 min) and 15, 30, 45, 60, and 120 min after injection, using the glucometer (ACCU-CHEK^®^ Aviva, Roche Diagnostics, Mannheim, Germany). The rate constant for glucose clearance (KITT) was calculated using the formula 0.693/t_1/2_ where t_1/2_ represents the half-life of plasma glucose decay. The plasma glucose t_1/2_ was calculated from the slope of the least squares analysis of the glycemic concentration during the linear phase of decay [48].

The area under the curve (AUC) of GTT (AUC_GTT_) and of ITT (AUC_ITT_) were calculated using the trapezoidal method [49].

### 2.5. Collection of Biological Samples 

At the end of the 14-week protocol, animals were euthanized with isoflurane overdose followed by cervical dislocation. Blood samples were immediately collected by venipuncture from the jugular vein and serum was obtained by centrifugation (3000× *g* for 15 min at 4 °C) and immediately stored at −20 °C until processing for biochemical analysis. The liver and small portions of gastrointestinal tissues (duodenum and colon) were immediately excised, dissected, and stored in conditions according to the assay’s technical requirements. The liver was firstly weighed and then divided into four distinct portions: a first piece was immediately used for functional mitochondria assays; the other two parts, reserved for protein and RNA extraction purposes, were directly frozen in liquid nitrogen and stored at −80 °C until analysis; and a fourth piece was kept in a 10% neutral buffered formalin solution to be used for histological analysis. The relative liver weight was calculated as the ratio of absolute tissue weight (g) to BW (kg). During the last week of experimental protocol, 24-h urine and fecal samples were collected using metabolic cages. During this period, rats had free access to water and food. The volume of urine was recorded; feces were weighed, and samples were stored at −80 °C for later analysis.

### 2.6. Measurement of Serum Metabolic Parameters 

Serum samples were used to perform the following measurements, through automatic validated methods and equipment (Hitachi 717 analyzer, Roche Diagnostics GMBH, Mannheim, Germany), as previously described [50]: postprandial glucose, triglycerides (TGs), total-cholesterol (Total-C), low-density lipoprotein cholesterol (LDL-C), and high-density lipoprotein cholesterol (HDL-C) levels, as well as serum glutamic-oxaloacetic (GOT) and glutamic-pyruvic (GPT) transaminases concentrations. Hemoglobin A1c (HbA1c) levels were determined using the DCA 2000+ analyzer (Bayer Diagnostics, Barcelona, Spain), according to the manufacturer’ instructions. Serum insulin levels were determined by Enzyme-Linked ImmunoSorbent Assay (ELISA) using commercially available kits for rat samples from Mercodia (Uppsala, Sweden). Insulin resistance was evaluated by the homeostatic model assessment of insulin resistance (HOMA-IR) index, which was calculated as previously described [51], using the following formula: HOMA-IR index = [fasting glucose (mmol/L) × fasting insulin (µU/L)]/22.5. High-sensitivity C-reactive protein (hs-CRP) was assayed by using a rat-specific Elisa kit (MBS764381 from Mybiosource, San Diego, CA, USA) according to the manufacturer’ instructions.

### 2.7. Determination of Serum Total Antioxidant Status (TAS)

For determination of the serum TAS, ferric reducing antioxidant potential (FRAP) was performed as previously described [52], whereas the scavenging of 2,2′-Azino-bis(3-ethylbenzothiazoline-6-sulfonic acid) radical cation (ABTS^●+^) assay was employed as described by Gião et al. [53].

The ABTS^●+^ stock solution was prepared by reacting equal amount of 7 mM ABTS diammonium salt (Sigma-Aldrich, St. Louis, MO, USA) and 2.45 mM potassium persulphate (Merck, Damstadt, Germany). The reaction was developed for 16 h in the dark. Aliquots of serum samples (10 µL), diluted when needed, were added to 1 mL of ABTS^●+^ solution with an initial optical density (OD) of 0.70 ± 0.02 measured at 734 nm. After allowing the reaction to occur, the OD was recorded using an UV-Vis spectrophotometer (UVmini 1240, Shimadzu, Japan) and the results were calculated as inhibition percentage (IP) of ABTS^●+^, according to the equation ABTS^●+^ inhibition (%) = 100 − [(OD sample × DF)/OD ABTS] × 100, where OD sample indicates the sample absorbance following 6 min of reaction, DF is the dilution factor and OD ABTS refers to the initial absorbance of the diluted ABTS^●+^ solution. All measurements were performed in triplicate.

### 2.8. H Nuclar Magnetic Resonsance (NMR) Spectroscopy

Before NMR analysis, 180 µL of the serum samples were mixed with 45 µL of a phosphate buffered (0.2 M) sodium fumarate (10 mM) solution (99.9% ^2^D_2_O) that was used as internal standard (Sigma-Aldrich, St. Louis, MO, USA) and each sample was loaded into 3 mm NMR grade tubes for high resolution ^1^H NMR analysis.

NMR spectra were obtained with a 600 MHz (14.1 T) spectrometer (Agilent, Santa Clara, CA, USA) equipped with a 3 mm indirect detection probe with a z-gradient. 1D-^1^H cpmg (Carr-Purcell-Meiboom-Gill spin-echo pulse sequence) experiments with water pre-saturation were acquired (7.2 kHz spectral width, 0.1 s mixing time, 4 s relaxation delay with 3 s of water pre-saturation, 90° pulse angle, 3 s acquisition time and 128 scans at 298 K). Pulse durations and water saturation frequencies were optimized for each sample. Spectra were processed by applying exponential line broadening (0.3 Hz), zero-filling to 64 k, and manual phasing and baseline correction. Chemical shifts were internally referenced to fumarate (singlet at 6.50 ppm). 

Spectral assignments were based on matching the recorded spectra to the reference data available in public databases such as Human Metabolome Database (HMDM) [54]. 2D homonuclear total correlation spectroscopy (TOCSY) spectra were recorded for selected samples to help spectral assignment [55]. All metabolites were identified according to Metabolomics Standards Initiative (MSI) guidelines for metabolite identification [56] and the levels of identification are indicated in Supplementary Materials Table S1.

Processed 1D cpmg spectra were bucketed using one-point bucket (0.6–9.0 ppm, with signal-free, water, and fumarate regions excluded) using Amix Viewer (version 3.9.15, Bruker Biospin GmbH, Rheinstetten, Germany) and aligned using icoshift algorithm [57]. Resulting matrix was normalized by total spectral area included in the analysis. Multivariate statistical analysis was applied on unit variance scaled matrix (SIMCA 14, Umetrics, Sartorius Stedim Biotech, Gottingen, Germany). In order to identify clustering trends or outliers, principal component analysis (PCA) was used to provide the information on global data structure, and partial least square discriminant analysis (PLS-DA) was used to assess class separation and identify the main metabolites that contribute to the class discrimination. A 7-fold internal cross-validation of the PLS-DA model was used to provide the qualitative measure of predictive power (Q2) and to assess the degree of fit to the data (R2). Permutation test (*n* = 100) was also used to validate the PLS-DA model [58]. The corresponding PLS-DA loadings plot was obtained by multiplying the loading weight factors (w) by the standard deviation of the respective variable and was color-coded according to variable importance in the projection (VIP). Selected signals of chosen metabolites (VIP > 1) were integrated in normalization by ^1^H-NMR spectra for quantitative assessment of metabolite variations between the groups. 

Outliers were excluded based on the quality of the recorded NMR spectra according to the recommendations of MSI [56]. The difference between the means of the two groups was assessed using the *t* test (results reported at a confidence level of 95%).

### 2.9. Evaluation of Serum Lipopolysaccharides (LPS) Concentration

Serum endotoxin LPS concentration was quantified using a Pyrochrome Lisate Mix, a quantitative chromogenic reagent, diluted in glucashield buffer, which inhibits cross-reactivity with (1 → 3)-β-d-glucans (Associate of Cape Cod Incorporated, East Falmouth, MA, USA). Briefly, serum samples were diluted (1:10) in pyrogen-free water (LAL reagent water, W50-100, Lonza, Walkersville, MD, USA) and heated for 10 min at 70 °C. Samples and pyrochrome reagent (1:1) were incubated at 37 °C for 30 min and absorbance was read at 405 nm.

### 2.10. Extraction and Quantification of Gut Microbiota in Feces

#### 2.10.1. DNA Extraction from Stool 

Genomic DNA was extracted and purified from fecal samples using the NZY Tissue gDNA Isolation Kit (NZYtech, Lisbon, Portugal) according to the manufacturer´s protocol with slight modifications [59]. Briefly, fecal samples (170 to 200 mg) were homogenized in Tris-EDTA buffer solution (10 mM Tris/HCl; 1 mM EDTA, pH 8.0) and centrifuged at 4000× *g* for 15 min. The supernatant was discarded, and the pellet was resuspended in 350 μL of buffer NT1. After an incubation step at 95 °C for 10 min, the samples were centrifuged at 11000× *g* for 1 min. Then, 25 μL of proteinase K was added to 200 μL of the supernatant for incubation at 70 °C for 10 min. The remaining steps followed the manufacturer’s instructions. DNA purity and quantification were assessed with a NanoDrop spectrophotometer (Thermo Fisher Scientific, Wilmington, DE, USA).

#### 2.10.2. Real-Time PCR for Microbial Analysis of Stool

Real-time PCR was performed in sealed 96-well microplates using a LightCycler FastStart DNA Master SYBR Green kit and a LightCycler instrument (Hoffman-La Roche Ltd., Basel, Switzerland) as previously described [59]. The assay was performed in a 50 μL sample containing a reaction mixture of 20 ng of DNA, with 25 μL of SsoAdvanced Universal SYBR Green (Bio-Rad, Hercules, CA, USA), 5 μL of each primer, and 10 μL of water. Primer sequences (Sigma-Aldrich, St. Louis, MO, USA) used to target the 16S rRNA gene of the bacteria and the conditions for PCR amplification reactions are listed in Table 1. To verify the specificity of the amplicon, a melting curve analysis was performed via monitoring SYBR Green fluorescence in the temperature ramp from 60 °C to 97 °C. Data were processed and analyzed using the LightCycler software (Hoffman-La Roche Ltd., Basel, Switzerland). Standard curves were constructed using serial tenfold dilutions of bacterial genomic DNA, according to the data provided on the following webpage (http://cels.uri.edu/gsc/cndna.html). Bacterial genomic DNA (DSMZ, Braunschweig, Germany) was used as a standard. Genome size and the copy number of the 16S rRNA gene for each bacterial strain used as a standard were obtained from the NCBI Genome database (www.ncbi.nlm.nih.gov). Data are presented as the mean values of duplicate PCR analysis.

### 2.11. Fecal SCFAs and Organic Acids Determination 

Short-chain fatty acids (SCFAs) and organic acids (lactic and succinic acid) were measured using an Agilent 1200 series HPLC system with a refractive index—RI detector and with a UV detector. Approximately 200 mg of feces were dissolved in 1 mL of ultrapure water, homogenized in a “mixer” for 15 min, and centrifuged at 10,000× *g* for 10 min; the supernatants were collected and stored at −20 °C until analysis. Briefly, fecal samples were filtered through a 0.22 μm membrane filter (Orange Scientific, Braine-l’Alleud, Belgium) and injected (40 µL) directly into an HPLC System consisting of a LaChrom L-7100 pump (Merck-Hitachi, Darmstadt, Germany) and an ion exchange Aminex HPX-87H column (300 × 7.8 mm, BioRad Laboratories, Inc., Hercules, CA, USA), operated at 65 °C. The mobile phase used was 0.003 M solution of sulfuric acid at a flow rate of 0.6 mL/min. Data were collected and analyzed with a D7000 Interface (LaChrom, Merck-Hitachi, Fullerton, CA, USA) and using HPLC System Manager^®^ Software 3.1.1 (MerckHitachi, Fullerton, CA, USA). Peak identification was based on the relative retention times determined by injection of standard solutions. Quantification was performed using calibration curves. Fecal SCFAs concentrations were expressed as mean micromoles per gram wet weight.

### 2.12. Colon and Duodenum Analysis by Transmission Electron Microscopy (TEM) 

Duodenum and colon samples were immediately sectioned in small fragments of approximately 1 mm^3^ and fixed in 2.5% glutaraldehyde solution in 0.1 M phosphate buffer (pH = 7.2) for 2 h. Sequential post-fixation was performed in 1% osmium tetroxide, for 1.5 h, and 1% aqueous uranyl acetate, for 1 h in the dark. After rinsing with distilled water, samples were dehydrated in a graded acetone series (30–100%) and embedded in an Epoxy resine (Fluka Analytical, Sigma-Aldrich, Darmstadt, Germany). Ultrathin sections obtained with a Leica EM UC6 (Leica Co, Vienna, Austria) ultramicrotome were mounted on copper grids and stained with lead citrate 0.2% for 10 min. Observations were carried out on a TEM Tecnai G2 Spirit Bio Twin at 100 kV (FEI, Hillsboro, OR, USA), and images were processed using AnalySIS 3.2.

### 2.13. Immunohistochemical Staining 

Cross-sections (10 μm thickness) of rat colon were cut with a cryostat (Leica CM3050S, Nussloch, Germany). Colon cryosection were fixed with an acetone:methanol mixture (1:1) at 20 °C for 2 min and then rehydrated in phosphate-buffered saline (PBS) (3 × 5 min). After washing, sections were permeabilized with 0.5% Triton X-100 in PBS for 15 min and blocked for 40 min with 4% nonfat milk in 20 mM Tris, pH 7.2, and 150 mM NaCl. The sections were incubated with primary antibodies: rabbit polyclonal anti-ZO-1 (ab96587, Abcam, Cambridge, MA, USA) and mouse monoclonal anti-occludin (OC-3F10, 33-1500, Life Tecnologies, Carlsbad, CA, USA) in PBS containing 1% BSA overnight at 4 °C. After rinsing with PBS (3 × 5 min), the sections were incubated with the secondary fluorescent antibody Alexa Fluor 488-conjugated goat anti-rabbit IgG or Alexa Fluor 568-conjugated donkey anti-mouse IgG (1:200; Molecular Probes, Life Technologies, Paisley, UK) and 4′,6-diamidino-2-phenylindole (DAPI, nuclei dye), for 1 h at room temperature. After incubation, the sections were washed with PBS (3 × 5 min), and the slides were mounted using the Glycergel mouting medium (Dako, Carpinteria, CA, USA). Anti-ZO-1 and anti-occludin immunostaining samples were imaged using a confocal fluorescence microscope (LSM 710, Carl Zeiss, Gottingen, Germany).

### 2.14. Hepatic Histological Analysis

Hepatic tissue samples were fixed directly in 10% neutral buffered formalin solution and embedded in paraffin wax. Paraffin blocks were cut to sections of 5 μm using a microtome (HM325, Thermo Fisher Scientific, Waltham, MA, USA). Hematoxylin-eosin (H&E) staining was performed according to the manufacturer’s guidelines (Merck Millipore, Darmstadt, Germany). Digital images of tissue slices were captured using a Zeiss microscope Mod. Axioplan 2 (Zeiss, Jena, Germany). 

Oil Red O staining was performed on frozen liver sections (5 µm) previously fixed in 10% formalin for 5 min as previously described [60]. Briefly, slides were rinsed three times with absolute propylene glycol and then placed in 0.5% Oil Red O stain solution in propylene glycol for 30 min before being rinsed with 85% propylene glycol for 1 min and counterstained with hematoxylin. Thereafter, the slides were washed with distilled water and mounted with aqueous mounting medium (Sigma, St. Louis, MO, USA). Sections were observed with a Zeiss microscope Mod. Axioplan 2 (Zeiss, Jena, Germany).

### 2.15. Quantification of Hepatic Triglycerides

Hepatic triglycerides levels were measured using a Triglycerides Colorimetric Assay kit (1155010, Cromatest^®^, Linear Chemicals, Barcelona, Spain). Briefly, 50 mg of frozen tissue were homogenized in 1 mL of isopropanol using a potter Elvehjem homogenizer (ThermoFisher, Waltham, MA, USA). The homogenate was sonicated and then centrifuged at 1000× *g* for 5 min at 4 °C. Triglycerides were detected at 450 nm using an enzymatic-photometric analyzer (BIOTEK^®^, Synergy HT, Winooski, VT, USA).

### 2.16. Hepatic Mitochondria Bioenergetics 

Hepatic mitochondria were isolated in homogenization medium containing 250 mM sucrose, 10 mM HEPES (pH 7.4), 0.5 mM EGTA, and 0.1% fat-free bovine serum albumin (BSA) [61,62]. After homogenization of the minced blood-free hepatic tissue, the homogenate was centrifuged at 800× *g* for 10 min at 4 °C. The supernatant was spun at 10,000× *g* for 10 min at 4 °C to pellet mitochondria, which were re-suspended in a final washing medium. EGTA and BSA were omitted from the final washing medium, adjusted at pH 7.4. Mitochondrial integrity was evaluated by measuring citrate synthase activity (CS), in the presence and absence of detergent (93 ± 2.5% of intact mitochondria after isolation). CS serves as a measure for membrane integrity since citrate synthase is located in the inner mitochondrial membrane, and thus should not be present in suspensions of mitochondria with intact membranes. Protein content was determined by the biuret method calibrated with BSA [63].

#### 2.16.1. Mitochondrial Permeability Transition (MPT) 

Mitochondrial swelling was estimated by changes in light scattering, as monitored spectrophotometrically at 540 nm, as previously described [64]. Reactions were carried out at 25 °C and Ca^2+^ (20 nmol) was added to the preparation after the start of the experiment. The assays were started by the addition of mitochondria (1 mg) to 2 mL of swelling medium (200 mM sucrose, 10 mM Tris–MOPS, 1 mM KH_2_PO_4_ and 10 μM EGTA, pH 7.4) supplemented with 2 μM rotenone and 5 mM succinate. To confirm the relationship between membrane permeability transition (MPT) induction and mitochondrial swelling, cyclosporine A (0.25 μM, a known MPT inhibitor) was added to the mitochondrial preparation before the addition of calcium. All the experiments were performed in triplicate. 

#### 2.16.2. Mitochondrial Respiration (Oxygen Consumption)

Oxygen consumption of isolated mitochondria was polarographically monitored with a Clark oxygen electrode (Oxygraph, Hansatech Instruments Ltd., Cambridge, UK) as previously described [61]. Mitochondria (1 mg) were suspended under constant magnetic stirring, at 25 °C, in 1.4 mL of standard respiratory buffer containing 130 mM sucrose, 50 mM KCl, 5 mM MgCl_2_, 5 mM KH_2_PO_4_, 50 μM EDTA, and 5 mM HEPES (pH 7.4) and 2 μM rotenone. Mitochondria were energized with succinate (5 mM) and state 3 respiration was induced by the addition of ADP (200 nmol). After the phosphorylation of the ADP to ATP, respiratory rate became slower (state 4). The respiratory control ratio (RCR) was calculated by the ratio between the state 3 and the state 4 respirations and used as a parameter of mitochondrial integrity. The uncoupled respiration was also measured in the presence of carbonylcyanide-P-trifluoromethoxyphenylhydrazon (FCCP, 1 μM). FCCP is an ionophore that uncouples oxidative phosphorylation by inducing artificial proton permeability in the mitochondria, stimulating the maximum respiration rate. The ADP/O ratio was calculated by the ratio between the amount of ADP added and the O_2_ consumed during the state 3 respiration.

#### 2.16.3. Mitochondrial Membrane Potential (ΔΨ)

Mitochondrial membrane potential (Δψ) was estimated using an ion-selective electrode to measure the distribution of tetraphenylphosphonium (TPP^+^) as previously described [62,64] using an Ag/AgCl_2_ electrode as reference. The entrance of TPP^+^ in mitochondria was determined by TPP^+^ concentration decreasing in the medium, measured by electrode potential. Briefly, mitochondria (1 mg) were suspended by gentle stirring in 1.4 mL of the standard respiratory buffer (as in mitochondrial respiration) supplemented with 3 μM TPP^+^ and energized by adding 5 mM succinate. To avoid complex I contribution due to possible endogenous substrates contribution and to prevent retrograde electron flow from the ubiquinone pool back to complex I, 2 µM of rotenone, a complex I inhibitor was added. After the steady-state distribution of TPP+ occurred, ADP (200 nmol) was added to initiate the phosphorylative cycle. The electrode was calibrated with TPP^+^ assuming Nernstian distribution of the ion across the synthetic membrane, and ΔΨ is expressed in -mV. A matrix volume of 1.1 μL/mg protein was assumed. The measured parameters were membrane potential (-mV), lag phase (seconds), and repolarization (-mV). Respiratory rates and ΔΨ were simultaneously measured.

### 2.17. Gene Expression by Quantitative Real-Time PCR Analysis 

Total RNA extraction from flash frozen liver was performed with PureLink RNA Mini Kit (12183018A, Ambion, Thermo Fisher Scientific, Carlsbad, CA, USA) according to the manufacturer´s instructions. Total RNA extraction from colon tissue samples was performed using a Trizol protocol (93289, Sigma Aldrich; St. Louis, MO, USA), and stored overnight at −80 °C. RNA concentration from liver was determined by Experion Automated Electrophoresis Station (Bio-Rad, Hercules, CA, USA). The concentration of total RNA from colon samples was measured by Nano Chip^®^ kit in Agilent 2100 bioanalyzer (2100 expert software, Agilent Technologies, Walbronn, Germany). RNA integrity (RIN, RNAIntegrity Number) and purity (A260/A280) of all RNA samples were measured by Nano Chip^®^ kit in Agilent 2100 Bioanalyzer (2100 expert software, Agilent Technologies, Walbronn, Germany) and ND-1000^®^ spectrophotometer (NanoDrop Tecnhologies, Wilmington, DE, USA), respectively.

In the hepatic samples, reverse transcription into cDNA was carried out by using the iScript Select cDNA Synthesis Kit (Bio-Rad, Hercules, CA, USA) following the manufacturer’s instructions. The colonic cDNA was synthesized from RNA using a Xpert cDNA Synthesis Mastermix (GK81.0100, GRISP, Porto, Portugal) following the manufacturer´s instructions. cDNA samples were then stored at −20 °C until use. 

To analyze genes of interest in colon tissue, gene expression quantification was performed as previously described [47]. Real-time PCR were conducted with a SYBR Green real-time PCR kit (Bio-Rad, Hercules, CA, USA), following the manufacturer’s recommendations and gene specific-primers for ZO-1 (Tjp1, Unique Assay ID: qRnoCID0001801), occludin (ocln; Unique Assay ID: qRnoCID0005733, Bio-Rad, Hercules, CA, USA), and mucin-2 (muc2; Unique Assay ID: qRnoCID0003629 Bio-Rad), which were normalized with GeNorm algorithm, where gene stability was attained with glyceraldehyde 3-phosphate dehydrogenase (GAPDH) and hypoxanthine-guanine phosphoribosyltransferase (HPRT). The relative expression ratio of each of the target gene was computed on the basis of ∆∆Ct (2-∆∆Cp) values 

In liver tissue, a predesigned 96-well Fatty Liver panel (SAB Target List, R96; 10046947, Bio-Rad, Hercules, CA, USA) for SYBR^®^ Green detection (Bio-Rad, Hercules, CA, USA) were used following the manufacturer’s instructions. This array includes genes for insulin signaling, adipokines, the inflammatory response, apoptosis, and carbohydrate and lipid metabolism in the liver. Gene expression was performed by SYBR-Green-based real time quantitative PCR using a StepOnePlus PCR system (Applied Biosystems, Foster City, CA, USA). 

### 2.18. Statistical Analysis

Results are expressed as mean ± standard error of the mean (SEM). Data was compared and analyzed using Student’s unpaired *t* test for normally distributed data or the Mann Whitney test for non-normally distributed data. One-way or two-way ANOVA followed by Bonferroni post hoc test was used as appropriate. Repeated measures ANOVA, followed by Bonferroni post-hoc test, was used to compare glucose levels throughout the GTT and ITT assays. Values of *p* ˂ 0.05 were considered statistically significant. GraphPad Prism for Windows (Version 6.0, GraphPad Software) was used for all statistical analysis.

## 3. Results

### 3.1. Phenolic Composition of Blueberry Juice

The phenolic composition of BJ was evaluated using the UV-vis spectra obtained on-line from a PDA detector, after chromatographic separation. The chromatographic profile obtained for the BJ is represented in Supplementary Materials, Figure S1. The main classes of phenolic compounds detected were hydroxycinnamic acids (peaks 1 to 3) and anthocyanins (peaks 4 to 11). Peaks 1 to 3 exhibited spectra profiles characteristic of caffeic or ferulic acid derivatives, with UV maxima near 250 and 324 nm. The other chromatographic peaks (4 to 11) were identified as anthocyanins due to the presence of very characteristic spectra, with a peak between 240 and 280 nm (band II) and a strong visible peak between 450 and 560 nm.

### 3.2. BW and Energy Intake

BW remained unchanged following the sustained BJ consumption, despite the increased consumption of BJ (and consequent increase of carbohydrate load) that paralleled a higher urine output (*p* < 0.05). No statistical differences were recorded on solid food ingestion and total energy intake between groups (Table 2).

### 3.3. Glycemic and Insulinemic Profiles

After BB consumption, serum glucose and insulin levels were not significantly altered in both fasting and postprandial conditions (Table 3). In GTT assay, blood glucose levels were transiently higher in the BJ group soon after the intraperitoneal glucose challenge [at 15 and 30 min (*p* < 0.01 and *p* < 0.05, respectively)] but quickly recovered in the following time-points, comparable to the control group. Accordingly, no statistical changes were found in AUC values of GTT (*p* > 0.05) nor in peripheral insulin sensitivity surrogates (ITT, kITT, HOMA-IR) (Table 3).

### 3.4. Intestinal Microbiota and Gut Barrier Integrity

**Figure 1:**
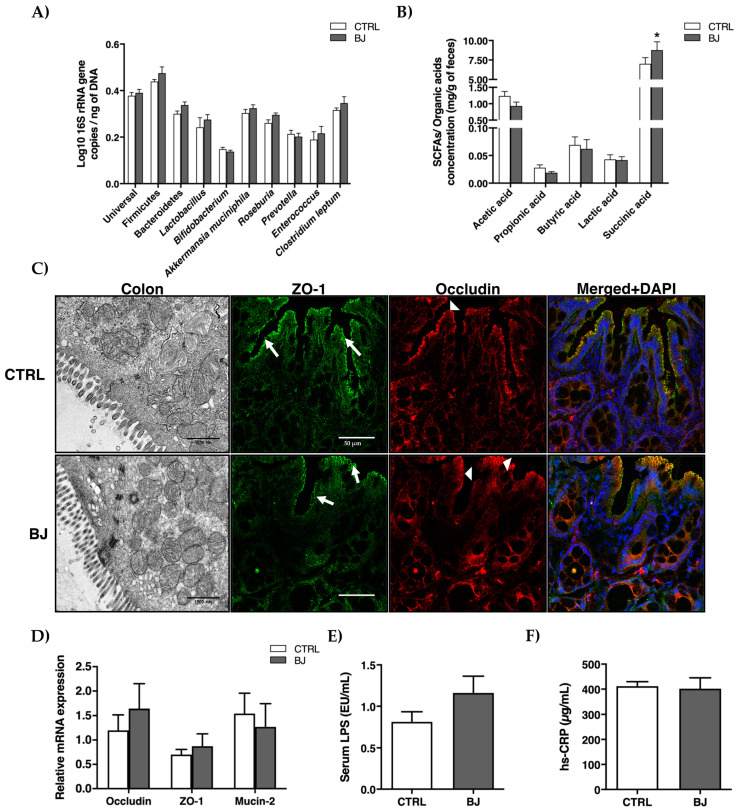
Gut microbiota composition (**A**), SCFAs, and organic acids (**B**) in feces; ultrastructural distribution of tight junctions and representative confocal images of colon section stained for ZO-1 (arrow) and occludin (arrowhead) (**C**) and mRNA expression (**D**) of key players of tight junctions; serum LPS (**E**) and hs-CRP (**F**) concentrations. Data are presented as mean ± SEM (*n* = 6–8/group); * *p* < 0.05 vs. CTRL group.

To assess the impact of a sustained BB consumption on GM composition and function, we next analyzed microbiota and SCFAs/organic acids composition in feces along with colonic ultra-structural morphology, intestinal permeability, and systemic inflammation. While fecal microbiota composition remained unchanged between the two groups (Figure 1A), a significant increase in succinic acid (*p* < 0.05) was detected in the BJ-treated rats (Figure 1B). Moreover, TEM analysis did not reveal any alteration in colonic barrier ultra-organization, namely in the intercellular junctions (Figure 1C), which was further corroborated by the expression of ZO-1, occluding, and mucin-2 genes, the three key players involved in the maintenance of epithelial integrity (Figure 1D). Correspondingly, serum endotoxemia (LPS) and inflammation (hs-CRP) remained unchanged in both experimental groups (Figure 1E,F).

### 3.5. Serum Antioxidant Status and Metabolomic Profile

**Figure 2:**
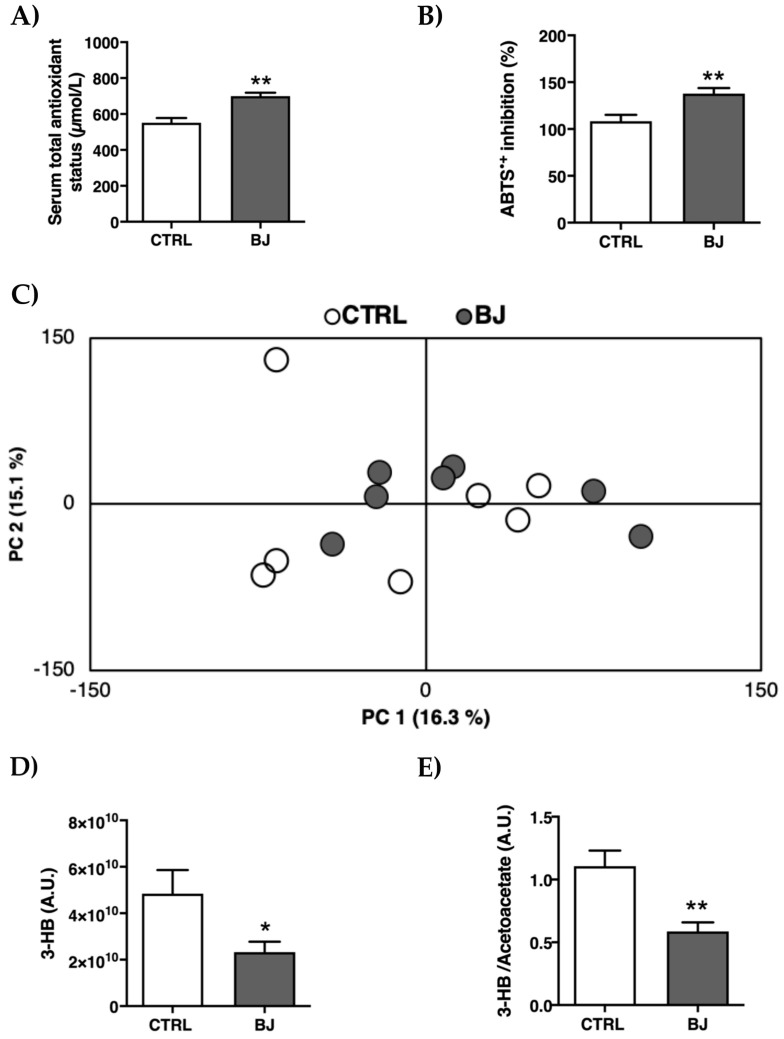
Serum antioxidant capacity evaluated by FRAP (**A**) and ABTS (**B**) assays; principal component analysis (PCA) scores plot obtained by multivariate analysis of ^1^H NMR spectra of serum data (**C**); Serum levels of 3-hydroxybutyrate (3-HB) (**D**) and 3-HB/acetoacetate ratio (**E**). Data are presented as mean ± SEM (*n* = 6–8/group); * *p* < 0.05 and ** *p* < 0.01 vs. CTRL group.

The serum antioxidant effect provided by this BJ consumption was evaluated by FRAP and ABTS^●+^ assays. As foreseen, BJ-treated rats presented higher serum TAS (*p* < 0.01, Figure 2A), along with increased serum ABTS^●+^ inhibition percentage (*p* < 0.01, Figure 2B).

To scrutinize the metabolomic profile between the control and BJ-supplemented animals, a nontargeted ^1^H NMR-based metabolic analysis was further performed, allowing for the simultaneous assessment of an array of serum metabolites. The resulting spectral profile identified 22 metabolites (Supplementary Materials, Table S1) without any global metabolic discrimination between groups (Figure 2C), notwithstanding the significant reduction (*p* < 0.05) of 3-HB and 3-HB/acetoacetate ratio in serum of BJ-supplemented rats (Figure 2D,E).

### 3.6. Serum and Hepatic Lipid Profile and Function

**Figure 3:**
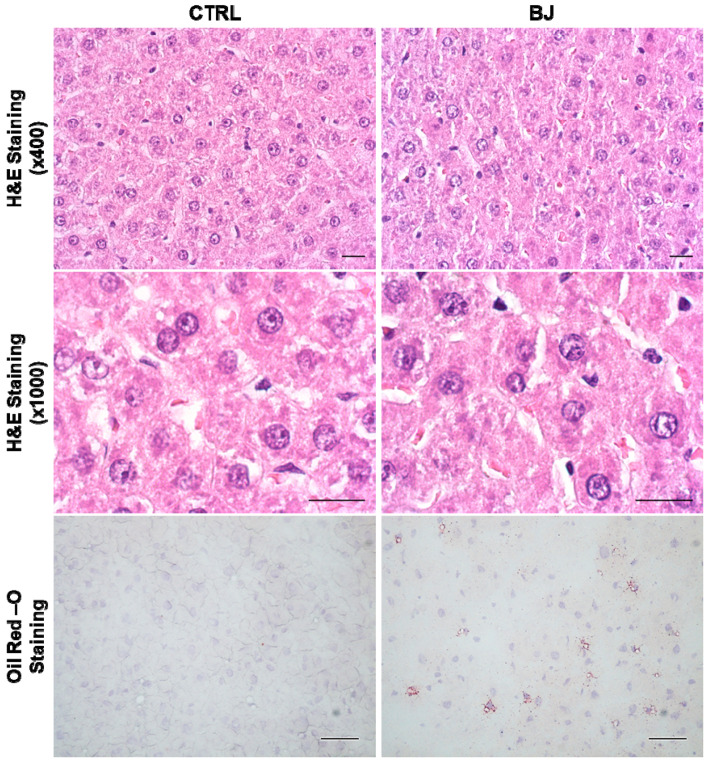
Representative images of hepatic structure and lipid accumulation evaluated by H&E (×400 and ×1000 magnification) and Oil-Red-O staining (×400 magnification), respectively, in the CTRL and BJ-treated rats; scale bar = 20 µm.

As shown in Table 4, comparable values were obtained for both groups regarding serum/hepatic lipid profiles as well as absolute liver weights. These results were substantiated by the regular histologic appearance without signs of lipid deposition observed in hepatic H&E and Oil-red-O stained sections, respectively (Figure 3). Likewise, similar serum GPT and GOT activities concurrently hint at a preserved liver function following BJ consumption (Table 4).

### 3.7. Hepatic Mitochondrial Bioenergetics

**Figure 4:**
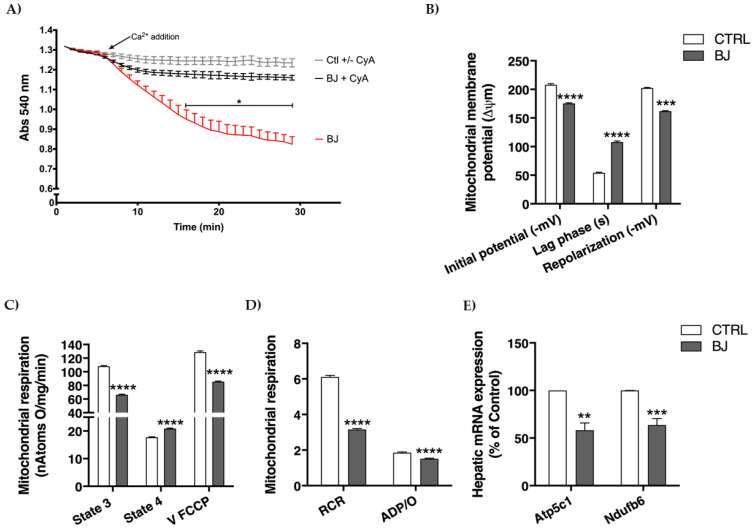
Hepatic mitochondrial function assessment: susceptibility to the induction of mitochondrial permeability transition (MPT) (**A**); mitochondrial membrane potentials and lag phase (**B**); parameters of mitochondrial respiration (**C**,**D**); hepatic mRNA expression of genes involved in mitochondrial respiratory chain (**E**). Data are presented as mean ± SEM (*n* = 6–8/group); ** *p* < 0.01, *** *p* < 0.001, **** *p* < 0.0001 vs. CTRL group.

In order to better understand the long-term effects of BB consumption on liver tissue, we assessed several parameters related with mitochondrial bioenergetics, an organelle with a crucial role in energy and metabolic regulation in the liver. Mitochondria have a limited capacity for accumulating calcium before undergoing the calcium-dependent mitochondria permeability transition (MPT), a phenomenon comprising the release of high molecular weight solutes from within the mitochondria, probably through the formation of a multi-channel pore. Mitochondrial swelling, an event that reveals mitochondrial permeability transition pore (mPTP) opening, was evaluated by presenting 20 nmol Ca^2+^ to mitochondrial preparations. In the presence of a relatively high Ca^2+^ dose, on pretreatment of hepatic mitochondria with cyclosporine A (MPT specific inhibitor), mitochondria retained aforesaid Ca^2+^ load and did not undergo Ca^2+^-dependent mitochondrial swelling, advocating isolated mitochondrial integrity (Figure 4A). Surprisingly, BJ induced a more pronounced decline in light scattering, indicating an increased susceptibility to undergo mitochondrial swelling, which will in turn reflect greater susceptibility to pore induction and mitochondrial uncoupling (Figure 4A).

Membrane potential (Δψ) sustained by mitochondria is central for this organelle function and pinpoints its phosphorylative capacity. As seen in Figure 4B, BJ induced a significant reduction in initial Δψ (after substrate addition) and in repolarization Δψ (mitochondrial capacity to establish Δψ after ADP phosphorylation) (*p* < 0.001). Moreover, the lag phase (time required for ADP phosphorylation), was significantly longer (*p* < 0.001) in hepatic mitochondria of BJ-supplemented animals. Mitochondrial respiration was further determined by evaluating oxygen consumption in energized mitochondria (succinate supply). BJ-isolated mitochondria showed a clear decline of respiratory capacity compared to the CTRL group. A decrease in mitochondrial state 3 (ADP-stimulated respiration) along with a significant increase in state 4 (resting state) respiration were observed (*p* < 0.001) in the mitochondria of BJ-treated rats (Figure 4C). Additionally, BJ supplementation also caused a significant decrease in uncoupled respiratory rate (V FCCP, a maximal respiratory activity stimulated by FCCP), suggesting that the phosphorylative system of hepatic mitochondria is impaired following a long-term BJ supplementation. Consistently, respiratory control ratio (RCR) was significantly decreased (*p* < 0.001) in hepatic mitochondria of BJ-treated animals. This functional parameter was determined by the ratio between state 3 and state 4 respiration and reflects the mitochondrial coupling between respiration and phosphorylation and efficiency. Moreover, the ADP/O ratio (which represents the number of ADP molecules that can be phosphorylated by one atom of oxygen consumed), a surrogate marker of mitochondrial oxidative phosphorylation efficiency, was also significantly decreased in BJ-treated rats (Figure 4D).

The expression of key genes encoding mitochondrial respiratory chain complexes was evaluated by RT-PCR. Interestingly, as shown in Figure 4E, both Atp5c1 and Ndufb6 were downregulated (*p* < 0.01) in animals supplemented with BJ when compared to the untreated CTRL animals. These two genes (Ndufb6 and Atp5c1) encode proteins of complex I (subunit NADH:ubiquinone oxidoreductase) and complex V (mitochondrial ATP synthase), respectively.

### 3.8. Hepatic RNA Transcripts Encoding Functionally Diverse Cellular Targets

A broad analysis of hepatic mRNA expression of BB phytochemical’s conceivable cellular targets, from chief-metabolic players to inflammatory components, was performed in both experimental groups.

**Figure 5:**
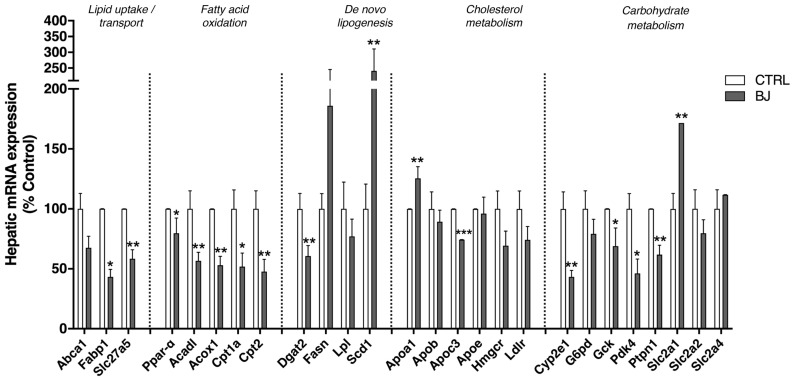
Hepatic mRNA expression of genes involved in fatty acid uptake/transport, fatty acid oxidation, and lipogenesis as well as cholesterol and carbohydrate metabolism. Data are presented as mean ± SEM (*n* = 5–6/group); * *p* < 0.05, ** *p* < 0.01, *** *p* < 0.001 vs. CTRL group.

The hepatic mRNA expression of genes involved in fatty acid uptake and transport, fatty acids oxidation, lipolysis, and synthesis (lipogenesis) were analyzed by qRT-PCR. BB consumption significantly decreased the hepatic mRNA expression of genes related with fatty acid transport (*Fabp1* and *Slc27a5*), fatty acid oxidation (*Ppar-α, Acadl*, *Acox1*, *Cpt1a*, *and Cpt2*), and lipid synthesis (*Dgat-2*) (Figure 5). In opposition, an enhanced expression of mRNA levels of *Scd1* (also lipogenic enzyme) (*p* < 0.005) was found in hepatic tissues. Yet, mRNA expression of some genes encoding lipogenic enzymes (*Fasn* and *Lpl*) did not show any significant alteration. Regarding cholesterol metabolism, a significantly increased mRNA expression of *Apoa1* (the major component of HDL) was found in the BJ group compared with the CTRL one (Figure 5). In addition, BJ supplementation significantly decreased mRNA expression of *ApoC3*, which encodes a small protein on the surface of VLDL and LDL. Hepatic alipoprotein (*Apo*) *B* and *Apo E*, *Hmgcr* and *Ldlr* mRNA expression were unchanged between experimental groups. In addition, the impact of BJ on cytochrome P450 2e1 (*Cyp2e1*) was also explored on the basis of its well-known influence in gluconeogenesis as well as in xenobiotic metabolism. Notably, *Cyp2e1* mRNA expression was significantly decreased in animals supplemented with BJ (Figure 5). The expression levels of genes involved in glucose metabolism were also assessed. *Gck*, *Pdk4*, and *Ptpn1* mRNA expression were downregulated in livers of BJ-treated rats despite normal *G6pd* mRNA expression (Figure 5). In addition, BJ treatment enhanced the *Slc2a1* mRNA level (corresponding to the gene encoding glucose transporter, GLUT1), while mRNA expression of *Slc2a2* and *Slc2a4* genes (encoding GLUT2 and GLUT4, respectively) were unchanged (Figure 5).

**Figure 6:**
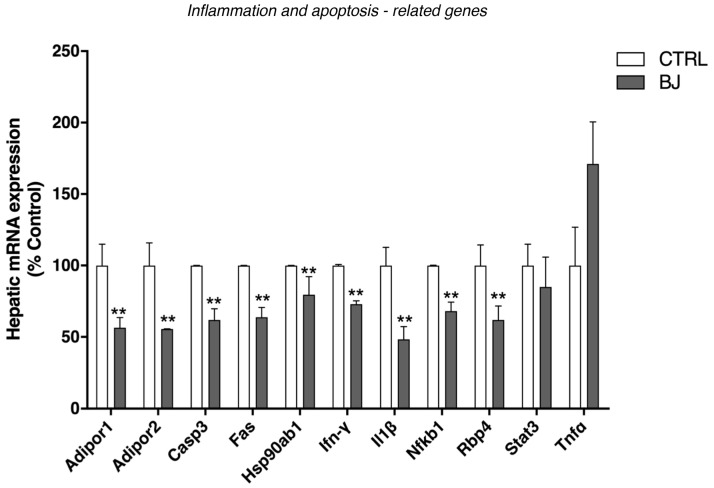
Hepatic mRNA expression of genes involved in inflammatory, stress response, and apoptotic processes. Data are presented as mean ± SEM (*n* = 5–6/group); ** *p* < 0.01 vs. CTRL group.

Due to the anti-inflammatory properties described for BB in several pathological conditions [65,66], we further underscored the expression of distinct inflammatory parameters. The mRNA expression of adiponectin receptors (*Adipor1* and *Adipor2*), tumor necrosis factor receptor superfamily member 6 precursor Tnfrsf6 (*Fas*), *Casp3*, *Ifn-γ*, interleukin-1 beta (*Il-1β*), *Nf-kB*, and *Rbp4* (gene that encodes a recently identified adipokine: retinol binding protein 4) were significantly lower in animals supplemented with BJ when compared to the CTRL group (Figure 6). Moreover, the hepatic expression of the heat shock protein (*Hsp90ab1*) was remarkably decreased in animals treated with BJ (Figure 6). No significant changes were found in the expression of the *Stat3* gene, a transcription factor involved in the downstream signaling of several cytokines and growth factors, as well as in the hepatic mRNA expression of tumor necrosis factor-α (*Tnf-α*) (Figure 6).

## 4. Discussion

Society globalization, time pressure, and the general concern surrounding a healthier lifestyle are major drivers to the rise of consumers’ demand for “superfoods” aimed to reinforce overall health status. Blueberries are classically tagged as “superfruits” on the basis of their richness in a panoply of bioactive phytochemicals throughout their outer and inner layers [6]. An emergent trend of BB “prophylactic” consumption is presently widely scaled-up, not only as a food source but also as commercially available formulations of BB-derived functional foods and supplements whose regulatory oversight varies from country-to-country. In this context, uncertainties regarding their “real-life” efficacy and possible adverse effects subsist [67,68,69]. Accordingly, reliable and reproducible information from preclinical and human trials is still incongruent and requires a more in-depth elucidation to guide a healthier and safer consumption [29,30,34,36,37]. Herein, we designed an experimental study in healthy Wistar rats that were exposed to a BB supplementation regimen (BB whole-fruit). Particular attention was given to the dose employed and treatment duration mirroring a long-term human consumption scenario, and gut- and liver-related metabolic parameters were assessed on the basis of BB-health benefit claims within obesity-related metabolic diseases.

We observed a moderate increase in fluid and carbohydrates consumption in BB-supplemented rats, most likely due to the pleasant organoleptic properties of BB fruits. Regardless of the fact that BJ may represent an extra source of carbohydrates, overall BB low caloric content along with the increased urine output observed in BB-supplemented rats favored a final isocaloric intake between groups and the maintenance of comparable BW growth curves. In fact, the lack of BW changes is in line with previous preclinical studies on healthy Sprague-Dawley and Lister hooded rats supplemented with freeze-dried whole BB for short- and long-term periods (3 and 7 weeks), respectively [70,71].

BB fruits are well-documented in regards to their glucose-lowering and insulin-sensitizing effects in several in vitro studies and in diabetic animal models [72,73,74,75,76,77]. In our study, BB-supplemented rats efficiently managed the higher carbohydrates load arising from BB consumption, without significant interference in global glycemic and insulinemic profiles, which is also in concordance with previous studies in healthy conditions [78,79,80].

Once orally consumed, BB phytochemicals are largely metabolized by resident gut microflora whose individual fingerprints distinctively shape bioactive compounds bioavailability [81]. Besides this chief role on BB phytochemicals kinetics, GM is also modulated by BB prebiotic molecules (e.g., dietary fibers and unabsorbed polyphenols) with recognized benefits in distinct metabolic diseased conditions [21,81,82,83]. Collectively, these reasons prompted us to characterize GM composition, SCFA-derived metabolites, and overall intestinal integrity once BB supplementation is orally provided. Our results do not depict any significant changes in microbiota composition, despite a significant increase in fecal succinic acid, an organic acid produced by bacterial fermentation of carbohydrates for which important roles on intestinal and hepatic gluconeogenesis as well as lipid metabolism are currently underlined [84,85,86]. Moreover, these observations are in line with studies of Haraguchi and colleagues, who reported enhanced cecal succinate levels upon consumption of some dietary polyphenols without major changes in propionate levels and succinate-producing gut bacteria (e.g., Bacteroidetes phylum) [87]. Albeit we acknowledge that modern analytical approaches available for microbiome assessment (e.g., 16S rRNA gene sequencing) could better discriminate subtle changes in GM upon BB supplementation [88], the increment in fecal succinic acid levels reported herein hints at a metabolic adaptative response of resident gut bacteria community towards enhanced fermentation of dietary fibers and intrinsic gut health-promoting effects [89,90,91]. The physiological levels of epithelial integrity modulators (e.g., occludin, ZO-1, mucin) along with a regular colonic ultrastructural morphology and the absence of systemic endotoxemia and inflammation further corroborates this assumption.

Blueberry’s health benefits are closely related with antioxidant activity [13,92,93] mainly due to their high content in phenolic compounds (e.g., hydroxycinnamic acids, caffeic or ferulic acid derivatives, anthocyanins), which we have verified in our BJ samples, analogous to what has been previously reported [94]. FRAP and ABTS^●+^ assays denoted a higher antioxidant capacity in serum samples of BB-supplemented rats, which is consistent with currently available preclinical and human data [95,96,97]. To decipher whether a long-term intake of BB could induce systemic metabolic effects, we conducted a non-targeted ^1^H NMR-based metabolomic approach. The resultant principal component analysis (PCA) did not discriminate any segregation between two experimental groups. Yet, an unexpected and marked decrease in serum 3-HB ketone bodies and 3-HB/acetoacetate ratio was observed in BB-supplemented rats. Circulating ketone body levels are main surrogates of energy metabolism and have been traditionally looked upon as an evolutionary ancient fasting energy vector from liver to peripheral tissues (e.g., brain, skeletal muscle, heart) [98]. Nevertheless, their importance as vital metabolic and signaling mediators has been recently underscored, even under regular glucose supply conditions [99].

Bearing in mind that (i) ketogenesis is primarily carried out in the hepatic mitochondria [100] and (ii) BB-derived phytochemicals (e.g., antioxidant polyphenols) are able to regulate mitochondrial metabolism/biogenesis and are tagged as mitochondria-protecting agents [101,102,103], we next attempted to dissect the impact of this BB consumption in hepatic tissue, with a major focus on mitochondrial function. Livers displayed a similar macroscopic appearance, regular histomorphology, and lipid deposition, which is aligned with the normal serum lipid profile observed between experimental groups. Moreover, the comparable serum GPT and GOT aminotransferases activities classically score for hepatic function preservation in a clinical perspective [104,105]. However, at a subcellular level, long-term BB intake clearly triggered a mitochondrial-adaptative setting, featured by an accentuated bioenergetic remodeling in isolated hepatic mitochondria. Mitochondrial respiration supports an electric potential (measured with the TPP^+^ electrode) and the decrease in this potential may result from an inhibitory effect at the level of the respiratory chain or due to an uncoupling effect (non-specific permeabilization of the membrane at the level of the internal membrane, or eventually the BJ has some ionophoretic property). These two effects can be simultaneous, and this is very likely, since respiratory state 4 rises significantly and V FCCP respiration is significantly inhibited. These two effects are reflected in an increase in the lag phase (the mitochondria take longer to phosphorylate the added ADP and do not phosphorylate it completely since the ADP/O lowers). Mitochondria are also important intracellular storage sites of the secondary messenger Ca^2+^, which accumulates in a direct relation with membrane potential. High concentrations of Ca^2+^ added to mitochondria in the presence of Pi promote the assemblage of a pore (permeability transition pore) that leads to loss of functional integrity of mitochondria (e.g., uncoupling) that cannot efficiently produce ATP.

In sum, the (i) mitochondrial swelling, a surrogate parameter of calcium-induced mPTP opening [106]; (ii) dissipation of the transmembrane electric potential (ΔΨ), probably reflecting proton leakage or inhibitory effects at the respiratory chain, or both [107,108]; (iii) overall impaired phosphorylative system, stemmed from the increased lag phase time (reflecting more time spent for ADP phosphorylation), decreased uncoupled respiration (V FCCP), lower RCR (implying an inefficient capacity for substrate oxidation and ATP turnover) and decreased ADP/O flux ratio [mol of ATP synthesized per mol of O (1/2 O_2_)] [109], collectively emphasize BB-derived phytochemicals ability to strongly impact mitochondrial respiratory control, in line with several reports focused on polyphenolic phytochemicals [110,111,112]. Moreover, a concomitant downregulation of mitochondrial respiratory complex I (Ndufb6) and complex V (Atp5c1) encoding genes further corroborates, at a molecular level, the mitochondrial functional data and is closely aligned with the dose-dependent decrease of ECT activity and oxidative phosphorylation observed in vitro when rat heart mitochondria were exposed to bilberry fruit anthocyanin-rich extracts [113].

To further underscore the first insights of BB-derived hepatic remodeling at a molecular level, we broadened our study through a transcriptomic analysis of functionally diverse cellular targets of BB phytochemicals encompassing energy-derived metabolic pathways, transcription factors, and inflammatory mediators. From the lipidic perspective, BB oral consumption triggered a pair-decreased mRNA transcripts of fatty acid transporter 5 (FATP5/Slc27a5) and fatty-acid binding protein-1 (FABP1), impaired hepatocyte fatty acid uptake, along with a collective transcriptional repression of Acadl, Acox-1, Cpt1a and Cpt2a encoding-genes, suggesting mitochondrial acyl-CoA uptake decline and successive fatty acid β-oxidation deceleration, which is in accordance with previously published data [114]. Interestingly, a concerted downregulation of PPAR-α gene, a transcription factor responsible for the induction of fatty acid transport/oxidation along with ketone biosynthesis and import, was also recorded. Taking into consideration that hepatic ketogenesis is a proxy of total hepatic fat oxidation [115,116], future work will be critical to better understand the possible correlation between the transcriptomic fatty acid β-oxidation repression and the decreased circulating 3-HB contents detected in BB-treated rats. Ensuing inhibition of the expression of the lipogenic enzyme DGAT-2 may also be postulated as a downstream event of PPAR-α transcription factor repression [117]. In opposition, the SCD-1 gene, which encodes an enzyme that catalyzes the conversion of saturated into monounsaturated fatty acids, was found up-regulated in orally supplemented BB rats. Furthermore, a simultaneous up- and downregulation of ApoA1 and ApoC3 gene transcription, two major structural components of HDL and VLDL lipoproteins, respectively, were detected in the livers of BB experimental group without a significant translation in serum/liver lipid profile. Within the carbohydrate context, BB-induced transcriptomic remodeling also followed similar traits. Glucokinase (GCK), the principal hexokinase acting as the gatekeeper for hepatic glycolysis, was found downregulated upon BB-supplementation [118,119]. A similar fall in Pdk4 gene transcription (probably due to PPAR-α downregulation) was also observed and may correlate with decreased pyruvate availability for gluconeogenic processes [118,119,120]. This assumption is further corroborated by CYP2E1 gene repression for which an important role in the oxidative metabolism of gluconeogenic substrates is well-known [121,122]. Moreover, Ptpn1 gene downregulation is compatible with insulin signaling improvement and is aligned with previous reports denoting the ability of polyphenol anthocyanins to exert their insulin-sensitizing properties [94,123]. Collectively, BB-induced hepatic transcriptomic metabolic reprogramming observed herein hints at a repression of chief fuel sources and cellular energetic pathways (e.g., fatty acid β-oxidation, glycolysis, gluconeogenesis).

Finally, and on the basis of the anti-inflammatory potential of BB phytochemicals [124,125], we also assessed the transcriptomic profile of a panoply of inflammatory-related genes. A combined gene repression of NFkb1, cytokines (e.g., IFN-g, IL-1β), adipokines (e.g., Adipor1 and Adipor2), and stress-element responses (e.g., Hsp90ab1) were unequivocally triggered by the BB supplementation. Besides their well-known antioxidant properties, BB phytochemicals also display the ability to modulate cellular pathways through epigenetic mechanisms encompassing DNA methylation, histone modifications, and posttranscriptional gene regulation of noncoding RNAs, constituting a link between external environmental cues and gene expression [4,126]. In this regard, we cannot overlook the probable “noncanonical” signaling rearrangement arising from BB phytochemicals-altered ketone bodies homeostasis. In fact, decreased 3-HB levels may also trigger chromatin remodeling through histone hypoacetylation and subsequent transcriptional repression [115,116]. Due to its reversibility, dietary-targeted epigenetics is an attractive approach for disease prevention and is often envisaged as a starting point of clinical intervention [127]. Taken together, and on the basis of the marked transcriptomic repression in metabolic, transcription factors, and inflammatory-related genes, future experimental studies are warranted to underscore the impact of long-term intake of BB on the epigenetic phenomena and their potential for disease prevention and/or intervention.

One emergent mechanism of action of dietary phytochemicals relies on their ability to act as hormetins, triggering adaptive cellular stress response pathways in both plants and humans [128]. Typically, the concept of hormesis has been adopted to describe the phenomenon where a given substance/condition is able to induce biologically opposite effects at different doses [129]. In other words, a hormetic response may occur when a mild stress (e.g., calorie restriction, exercise) activates intrinsic changes that enhance resistance to a more severe stress arising from higher doses of the same stressor or even from other less-specific stressors comprising oxidative, metabolic, and thermal stresses [130]. Altogether, the mitochondrial bioenergetic challenge and metabolic transcriptomic reprogramming presently disclosed portray a unified adaptive response to the nutritional challenge imposed by BB phytochemicals intake. Interestingly, mitohormesis is being widely disclosed in many model organisms and strongly implicated in metabolic health [131,132,133,134]. For instance, mitochondrial ECT modulation was found to reprogram energy metabolism towards cell survival and improved lifespan [135]. Remarkably, downregulation of ECT activity due to mitochondria-targeting xenobiotics in Caenorhabditis elegans resulted in cytoplasmic proteostasis restoration and suspected increased vitality later in life [136]. Moreover, mitochondrial bioenergetics remodeling may also encompass a central node in the fine-tuning of metabolic “switch” in fuel sources and catabolic/anabolic rates that optimize organism performance in varying nutrient states and physiological conditions [116,137]. Interestingly, the global repression of genes enclosing distinct metabolic pathways collectively hint for a “hibernated” energetic state upon BB supplementation. Conceivable altered ketogenesis, classically viewed as a spillover pathway from fat combustion, further reinforces a quiescent mitochondrial-related metabolomics. Supported by the notion that ketone body metabolism may be beneficial even in carbohydrate-laden states, the ketohormetic hypothesis has been recently postulated [116] and may actually intertwine with mitohormesis [116,138] within this BB phytochemical challenge.

Since the hormetic response is a phenomenon where low doses of a stressor trigger opposite responses to high ones, the beneficial or toxic outcomes should be interpreted in the light of the non-linear biphasic hormetic dose-effect relationship [139]. Accordingly, the amounts of phytochemical cell stressors in fruits and vegetables consumed by humans in a regular diet are considered to fall within the low dose stimulatory range of concentrations; however, once they are consumed in the form of concentrated supplements, the doses may exceed the toxic threshold with potential adverse health consequences [130,140]. Remarkably, previous work also disclosed that hepatic tissue is able to hormetically cope with chemical stressors (ethanol) through a transcriptional adaptative response [141]. Considering that we only assessed a unique dose in a single-hit stress paradigm (BB consumption) along with the ambivalent redox nature of polyphenols [110], the beneficial or noxious consequences arising from this BB hepatic remodeling should be carefully interpreted. In a global perspective, no evident toxic effects were recorded. However, at a molecular level, BB intake may trigger distinct consequences. For instance, if the transcriptomics remodeling of some of the analyzed genes are effectively translated into the protein level, one can envisage both positive [anti-atherogenic properties (e.g., ApoA1 upregulation) and hepatic steatosis reversal (e.g., SCD-1 upregulation)] and negative [altered xenobiotic metabolism (e.g., acetaminophen, isoniazid, coumarin, ethanol), due to Cyp2E1 downregulation] outcomes [142,143,144]. Moreover, the mitochondrial “idle motion” observed herein may be beneficial at a basal condition and prelude optimal fitness but also be detrimental if not quickly reversed upon highly demanding operating challenges.

## 5. Conclusions

To the best of our knowledge, this is the first work providing molecular insights on the mitochondrial and metabolic effects of BB consumption in a healthy condition from a translational perspective. Collectively, the data presented herein hint at mitochondrial-related metabolic transcriptomic reprogramming, together with a concerted anti-inflammatory pre-conditioning. Future studies comprising prophylactic or interventional BB regimens in diseased conditions (two-hit experimental paradigms) and in both sexes will be of utmost importance to disclose tailored beneficial/toxic outcomes of this new dietary trend and guide evidence-based medicine.

## Figures and Tables

**Table 1:** Primer sequences and real-time PCR conditions used for gut microbiota analysis.

Bacterial Group	Primer Sequence (5′-3′)	PCR Product Size (bp)	AT (°C)
Firmicutes	ATG TGG TTT AAT TCG AAG CA	126	45
	AGC TGA CGA CAA CCA TGC AC		
Bacteroidetes	CAT GTG GTT TAA TTC GAT GAT	126	45
	AGC TGA CGA CAA CCA TGC AG		
Clostridium	GCA CAA GCA GTG GAG T	239	45
	CTT CCT CCG TTT TGT CAA		
Bacteroides	ATA GCC TTT CGA AAG RAA GAT	495	45
	CCA GTA TCA ACT GCA ATT TTA		
Universal	AAA CTC AAA KGA ATT GAC GG	180	45
	CTC ACR RCA CGA GCT GAC		
Enterococcus	CCC TTA TTG TTA GTT GCC GCC ATC ATT	144	50
	ACTCGT TGT ACT TCC CT TGT		
Prevotella	CAC RGT AAA CGA TGG ATG CC	513	50
	GGT CGG GTT GCA GAC C		
Bifidobacterium	CGC GTC YGG TGT GAA AG	244	50
	CCC CAC ATC CAG CAT CCA		
Roseburia	TAC TGC ATT GGA AAC TGT CG	230	50
	CGG CAC CGA AGA GCA AT		
Lactobacillus	GAG GCA GCA GTA GGG AAT CTT C	126	55
	GGC CAG TTA CTA CCT CTA TCC TTC TTC		
Akkermansia	CAG CAC GTG AAG GTG GGG AC	327	55
	CCT TGC GGT TGG CTT CAG AT		

**Abbreviations:** AT, annealing temperature; bp, base pairs; PCR, polymerase chain reaction.

**Table 2:** BW variation and cumulative energy intake during the experimental protocol.

Parameters		CTRL	BJ
Body weight	Initial (g)	461.00 ± 14.00	472.30 ± 14.61
	Final (g)	510.60 ± 20.85	532.40 ± 24.42
	Delta (g)	49.57 ± 8.71	60.13 ± 10.80
Intakes	Food (g/rat/week)	164.90 ± 2.43	162.50 ± 2.62
	Drink (mL/rat/week)	210.20 ± 6.42	323.40 ± 11.20 ***
	Total calories (Kcal/rat/week)	519.30 ± 7.65	542.90 ± 8.51
	Carbohydrates (Kcal/rat/week)	352.70 ± 5.89	382.20 ± 5.77 ***
	Lipids (Kcal/rat/week)	44.52 ± 0.66	43.16 ± 0.67
	Proteins (Kcal/rat/week)	122.00 ± 1.80	118.80 ± 1.95
Urine Output	Urine volume (mL/day)	19.43 ± 2.68	42.25 ± 6.41 **

Data are presented as mean ± SEM (*n* = 8 per group). ** *p* < 0.01 and *** *p* < 0.001 vs. CTRL group.

**Table 3:** Glycemic and insulinemic profiles.

Parameter	CTRL	BJ
Fasting glucose (mg/dL)	101.30 ± 2.29	99.50 ± 2.26
Postprandial glucose (mg/dL)	149.30 ± 9.40	159.00 ± 11.15
Glucose levels (mg/dL) during GTT		
0 min after glucose load	100.9 ± 1.53	99.71 ± 2.32
15 min after glucose load	314.4 ± 21.98	412.10 ± 14.19 **
30 min after glucose load	342.00 ± 37.55	428.40 ± 6.121 *
60 min after glucose load	248.10 ± 30.30	289.70 ± 16.01
120 min after glucose load	199.30 ± 18.14	185.60 ± 7.81
AUC_GTT_	30,313.00 ± 2887.00	35,174.00 ± 868.70
Fasting insulin (mU/L)	0.65 ± 0.05	0.61 ± 0.11
Postprandial insulin (mU/L)	1.02 ± 0.21	1.24 ± 0.15
Glucose levels (mg/dL) during ITT		
0 min after insulin injection	100.80 ± 1.82	99.50 ± 2.79
15 min after insulin injection	97.57 ± 4.36	103.10 ± 4.58
30 min after insulin injection	74.57 ± 2.37	80.25 ± 4.97
45 min after insulin injection	60.57 ± 2.06	67.38 ± 3.91
60 min after insulin injection	58.86 ± 2.52	63.25 ± 2.58
120 min after insulin injection	55.43 ± 5.59	64.50 ± 5.26
AUC_ITT_	8120.00 ± 291.70	8815.00 ± 317.20
kITT (% min)	0.68 ± 0.06	0.52 ± 0.06
HOMA-IR	3.26 ± 0.60	3.86 ± 0.72

Data are presented as mean ± SEM (*n* = 6/8 per group). AUC, area under curve; GTT, glucose tolerance test; HOMA-IR, homeostatic model assessment of insulin resistance; ITT, insulin tolerance test; kITT, glucose disappearance rate for ITT; * *p* < 0.05 and ** *p* < 0.01 vs. CTRL group.

**Table 4:** Serum lipid profile and hepatic parameters.

Parameter	CTRL	BJ
**Serum lipid profile**		
TGs (mg/dL)	130.00 ± 16.34	153.10 ± 16.09
Total-C (mg/dL)	71.25 ± 3.37	72.25 ± 5.28
LDL-C (mg/dL)	20.75 ± 1.51	19.50 ± 1.19
HDL-C (mg/dL)	44.75 ± 1.99	44.63 ± 7.46
TGs/HDL-C ratio	3.04 ± 0.37	3.65 ± 0.54
**Hepatic parameter**		
Liver weight (g)	13.20 ± 0.44	14.28 ± 0.69
Liver weight/BW (g/kg)	25.94 ± 0.62	26.85 ± 0.55
TGs (mg/g tissues)	12.34 ± 0.88	12.04 ± 0.49
GPT (U/L)	35.50 ± 1.96	36.29 ± 2.39
GOT (U/L)	67.13 ± 3.12	68.29 ± 6.30

Data are presented as mean ± SEM (*n* = 6/8 per group). BW, body weight; GPT, glutamic-pyruvic transaminase; GOT, glutamic pyruvic transaminase; HDL-C, high-density lipoprotein cholesterol; LDL-C, low-density lipoprotein cholesterol; TGs, triglycerides; Total-C, Total-cholesterol.

## Supplementary Materials

The following are available online at https://www.mdpi.com/1999-4923/12/11/1094/s1, Figure S1: Chromatographic profile of phenolic compounds in BJ, obtained with HPLC-PDA (320/530 nm), Table S1: Serum metabolites identified by 1H-NMR.
