# Supplementary material for: Blueberry Consumption Challenges Hepatic Mitochondrial Bioenergetics and Elicits Transcriptomics Reprogramming in Healthy Wistar Rats
Source: Pharmaceutics. 2020 Nov 14;12(11):1094. doi: 10.3390/pharmaceutics12111094 (PMC7697217; doi:10.3390/pharmaceutics12111094)
Supplement: Supplementary file 1 [file pharmaceutics-12-01094-s001.pdf]

pharmaceutics

Supplementary Materials: Blueberry Consumption Challenges Hepatic Mitochondrial Bioenergetics and Elicits Transcriptomics Reprogramming in Healthy Wistar Rats

Sara Nunes, Sofia D. Viana, Inês Preguiça, André Alves, Rosa Fernandes, João S. Teodoro, Artur Figueirinha, Lígia Salgueiro Sara Silva, Ivana Jarak, Rui A. Carvalho, Cláudia Cavadas, Anabela P. Rolo, Carlos M. Palmeira, Maria M. Pintado and Flávio Reis

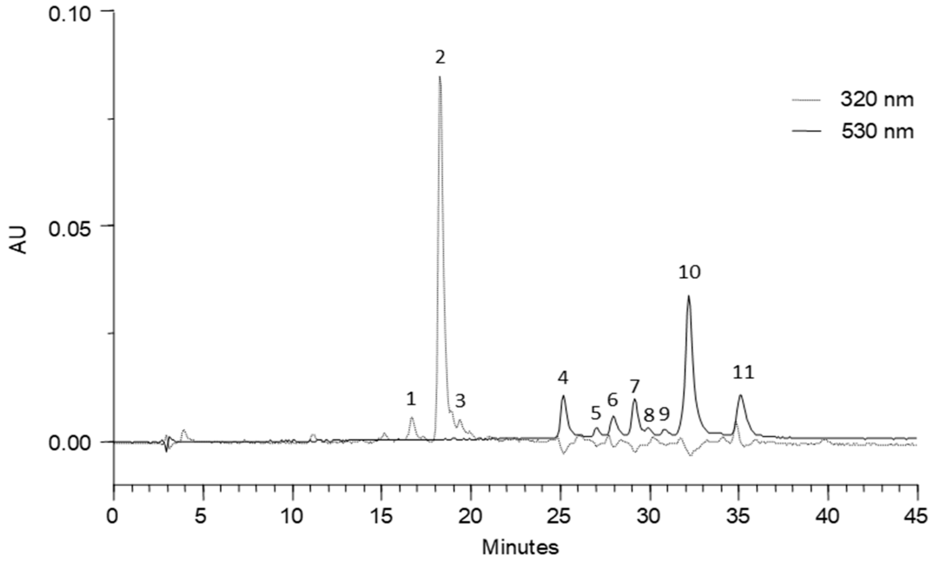

Figure S1. Chromatographic profile of phenolic compounds in BJ, obtained with HPLC-PDA (320 / 530 nm).

Table S1. Serum metabolites identified by ^{1}H-NMR.

Serum metabolites	Chemical shift (ppm)	CTRL (n=7)	BJ (n=7)
Histidine	7.010–7.070	$0.001 \pm 1.15 \times 10^{-4}$	$0.001 \pm 1.10 \times 10^{-4}$
TGs	5.230 (5.244–5.340)	$0.010 \pm 1.56 \times 10^{-3}$	$0.010 \pm 8.72 \times 10^{-4}$
Glucose	5.189–5.235	$0.026 \pm 1.13 \times 10^{-3}$	$0.026 \pm 8.94 \times 10^{-4}$
Mannose	5.149–5.176	$0.001 \pm 5.90 \times 10^{-5}$	$0.001 \pm 1.00 \times 10^{-4}$
Malic acid	4.261–4.307	$0.010 \pm 1.96 \times 10^{-3}$	$0.014 \pm 9.82 \times 10^{-4}$
Serine	3.947–3.985	$0.004 \pm 3.86 \times 10^{-4}$	$0.004 \pm 3.16e \times 10^{-4}$
Glycerol	3.640–3.635	$0.006 \pm 6.35 \times 10^{-4}$	$0.005 \pm 3.96 \times 10^{-4}$
Glycine	3.538–3.544	$0.007 \pm 8.81 \times 10^{-4}$	$0.007 \pm 8.89 \times 10^{-4}$
Choline	3.175–3.184	$0.005 \pm 9.86 \times 10^{-4}$	$0.004 \pm 7.31 \times 10^{-4}$
unknown (DMSO?)	3.124–3.134	$0.001 \pm 1.19 \times 10^{-4}$	$0.001 \pm 1.20 \times 10^{-4}$
Creatine	3.005–3.023	$0.003 \pm 4.16 \times 10^{-4}$	$0.004 \pm 4.22 \times 10^{-4}$
Glutamine	2.406–2.457	$0.016 \pm 9.70 \times 10^{-4}$	$0.017 \pm 1.29 \times 10^{-3}$
Succinate	2.379–2.387	$0.003 \pm 3.92 \times 10^{-4}$	$0.002 \pm 4.54 \times 10^{-4}$
Acetoacetate/Acetone[a]	2.195–2.217	$0.003 \pm 5.96 \times 10^{-4}$	$0.003 \pm 5.06 \times 10^{-4}$
N-acetylproteins	2.004–2.045	$0.016 \pm 9.96 \times 10^{-4}$	$0.017 \pm 9.40 \times 10^{-4}$
Acetate	1.889–1.904	$0.003 \pm 3.56 \times 10^{-4}$	$0.002 \pm 1.20 \times 10^{-4}$
Alanine	1.457–1.475	$0.007 \pm 3.93 \times 10^{-4}$	$0.008 \pm 4.09 \times 10^{-4}$
Lactate	1.292–1.334	$0.083 \pm 8.06 \times 10^{-3}$	$0.073 \pm 5.70 \times 10^{-4}$
3-hydroxybutyrate	1.163–1.189	$0.003 \pm 7.42 \times 10^{-4}$	$0.002 \pm 3.29 \times 10^{-4}$*
Valine	1.004–1.035	$0.006 \pm 2.98 \times 10^{-4}$	$0.006 \pm 5.70 \times 10^{-4}$
Isoleucine	0.997–1.001	$4.99 \times 10^{-5} \pm 8.5 \times 10^{-6}$	$4.03 \times 10^{-5} \pm 8.31 \times 10^{-6}$
TGs (0.87)	0.870 (0.825–0.893)	$0.010 \pm 1.71 \times 10^{-3}$	$0.015 \pm 1.66 \times 10^{-3}$
Betaine	3.260	$1.70 \times 10^{11} \pm 2.34 \times 10^{10}$	$1.21 \times 10^{11} \pm 1.47 \times 10^{10}$
Lactate /Alanine		13.190 ± 0.932	9.800 ± 1.221
3-HB / Acetoacetate		1.107 ± 0.124	0.587 ± 0.072**

Data are presented as mean ± SEM (n = 5–6/group). * $p < 0.05$, ** $p < 0.01$ vs. CTRL group. [a] Putatively annotated as level 3 of identification according to Chemical Analysis Working Group (CAWG) Metabolomics Standards Initiative recommendations. All other metabolites identified as level 2.
